# Cooler and drier conditions increase parasitism in a subtropical damselfly population

**DOI:** 10.1002/ece3.10897

**Published:** 2024-01-31

**Authors:** Shatabdi Paul, Mostakim Rayhan, Marie E. Herberstein, Md Kawsar Khan

**Affiliations:** ^1^ School of Natural Sciences Macquarie University Sydney New South Wales Australia; ^2^ Department of Biochemistry and Molecular Biology Shahjalal University of Science and Technology Sylhet Bangladesh; ^3^ Department of Biology, Chemistry and Pharmacy Freie Universität Berlin Berlin Germany

**Keywords:** climate change, host–pathogen interactions, insect decline, seasonal variation

## Abstract

Host–parasite interactions are impacted by climate, which may result in variation of parasitism across landscapes and time. Understanding how parasitism varies across these spatio‐temporal scales is crucial to predicting how organisms will respond to and cope under a rapidly changing climate. Empirical work on how parasitism varies across climates is limited. Here, we examine the variation of parasitism across seasons and identify the likely climatic factors that explain this variation using *Agriocnemis femina* damselflies and *Arrenurus* water mite ectoparasites as a host–parasite study system. We assessed parasitism in a natural population in Sylhet, Bangladesh which is located in subtropical climate between 2021 and 2023. We calculated prevalence (proportion of infected individuals) and intensity (the number of parasites on an infected individual) of parasitism across different seasons. Parasite prevalence and intensity were greater during cooler seasons (autumn and winter) compared to hotter seasons (spring and summer). Mean temperature and precipitation were negatively correlated with parasite prevalence, whereas only mean precipitation was negatively correlated with parasite intensity. Tropical, subtropical and mediterranean regions are predicted to experience extreme climatic events (extreme temperature, less precipitation and frequent drought) as a consequence of anthropogenic climate change, and our finding suggests that this might alter patterns of parasitism in aquatic insects.

## Graphical abstract

Understanding temporal variation of host–pathogen interactions might help to understand the impact of climate change on insect diseases. Here, we showed that seasonal variation of temperature and precipitation drives parasitism in subtropical climate. Our study highlights that climate change might increase diseases in subtropical climate which may contribute to further insect decline in the subtropics.

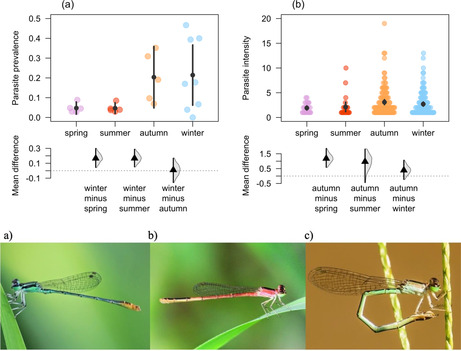

## 1 INTRODUCTION

Host–pathogen interactions are impacted by the environment in which they occur (Kiewnick, 2006; Poisot et al., 2017). Local climate such as temperature, precipitation, as well as resource availability and predator–prey interactions impact host immunity and pathogen virulence (Friman et al., 2009; Hassall et al., 2010; LoScerbo et al., 2020). Consequently, the outcome of host–pathogen interactions, that is, infections, varies across different climatic conditions. For example, in *Eulamprus quoyii* lizard, parasite intensity was greater in tropical climate compared to temperate climate (Salkeld et al., 2008). Similar to latitudinal variation, parasitism also varies across seasons mostly driven by the variation of seasonal temperature (Hassall et al., 2010; Zamora‐Vilchis et al., 2012). For example, in fire ants (*Solenopsis invicta*) parasite infections were greater in summer (Valles et al., 2010). Seasonal change in rainfall, on the other hand, is the strongest predictor of parasite infection in aquatic and semi‐aquatic organisms (Majumder et al., 2015; Zemmer et al., 2017). For example, in freshwater snails (e.g. *Elimia proxima*) and fish (e.g. *Hoplias malabaricus* and *Cirrhinus mrigala*) parasitism was negatively related to precipitation (Gonçalves et al., 2016; Majumder et al., 2015; Zemmer et al., 2017). How parasitism varies across seasons and what climatic factors affect parasitism in aquatic and semi‐aquatic insects is, however, less understood, primarily because studies tend to focus on northern hemisphere temperate populations where insects are active only for a short period, with limited seasonal variation.

Damselflies (Odonata: Insecta) are semi‐aquatic insects with an aquatic larval stage and a terrestrial adult stage. They are frequently parasitised by *Arrenurus* (Trombidiformes: *Arrenuridae*) water mites that externally attach to their body and wings (Khan & Herberstein, 2022; Paul et al., 2022). The extent of parasitism varies between sexes, developmental stages and in different climates (LoScerbo et al., 2020; Paul et al., 2022). For instance, ectoparasite prevalence and intensity in odonates were greater in the temperate climates compared to the boreal climates of the Northern Hemisphere (LoScerbo et al., 2020). Research on seasonal parasitism in damselflies is limited, with one of the few studies reporting that the extent of ectoparasitism in *Coenagrion puella* was greater during late spring (May) and the cooler period of the summer season compared to the warmer period of the summer season (August), but temperature was not associated with the variation in parasite prevalence (Hassall et al., 2010). It is noteworthy that there is a significant knowledge gap about damselfly parasitism in tropical regions (da Silva et al., 2021) with the majority of studies focussing on temperate populations where the flight season is very short and variation of climatic factors is limited (da Silva et al., 2021). Yet, tropical insects (e.g. damselfly) are more vulnerable to climate change than temperate insects, and understanding the influence of tropical seasons and identifying the climatic drivers in parasitism is of high priority to predict how climate change might affect insect–parasite interaction (Shah et al., 2017).

Our study aims to understand the pattern and driver of seasonal variation in parasitism of *Agriocnemis femina* damselflies. We studied the prevalence and intensity of infection by ectoparasite water mite in the natural population of north‐eastern Bangladesh, where these damselflies are active throughout the year. Based on previous studies in the Northern Hemisphere, we predict that (1) parasitism will vary over the season, (2) parasitism will be lower in summer when temperature and precipitation is higher compared to winter greatest.

## 2 METHODS AND MATERIALS

### 2.1 Study system

*Agriocnemis femina* (Coenagrionidae) is the one of the smallest damselflies (wing length: 10.5–11.00 mm) and occurs in South Asia, South‐East Asia and Australia (Kalkman et al., 2020; Orr et al., 2021; Shah & Khan, 2020) (Figure S1). This species is commonly found in grassland associated with water bodies such as ponds, lakes and rivers. Female *Agriocnemis femina* exhibit ontogenetic colour change from red to green, which signals sexual maturity (Khan, 2020). *Agriocnemis femina* is one of the most common species in the north‐eastern region of Bangladesh and can be seen in flight all year round (Shah & Khan, 2020). This species is parasitised by *Arrenurus* water mites (Paul et al., 2022). These mites are aquatic invertebrates which live in freshwater and their shapes vary from rounded to elongate (Smith, 1988). *Arrenurus* water mites have 3 pairs of legs as larvae with spine‐like setae and swimming hairs (Smith, 1988). Larval water mites initially colonise the aquatic damselfly larvae and then shift to the adult during damselfly metamorphosis on which they commence the parasitic phase and use chelicerae (mouth parts) to extract host body fluid (Smith, 1988), imposing considerable fitness costs on the host (Braune & Rolff, 2001; Khan & Herberstein, 2022).

### 2.2 Study site

We surveyed parasitism in *Agriocnemis femina* damselflies in the north‐eastern region of Bangladesh in a natural population located on the campus of Shahjalal University of Science and Technology, Bangladesh. The area of the study site is approximately 450 m^2^ and its perimeter is approximately 230 m. The study site is a small pond surrounded by agricultural lands. The pond is permanent with stagnant water flow, but the pond and surrounding areas experience flash floods during monsoons. Spring and summers of this study region are hot (average spring and summer temperature: 25.8 and 28.25°C respectively) with high rainfall (average spring and summer rainfall: 328.6 and 695.3 mm respectively) (Fick & Hijmans, 2017). Autumn and winter are comparatively colder (average autumn and winter temperature: 25.8 and 18.5°C respectively) and experience less rainfall (average autumn and winter rainfall: 244.3 and 21.5 mm) (Fick & Hijmans, 2017).

We surveyed the study site every month from March 2021 to February 2023 (except in July 2021, February–June 2022 and September–October 2022 due to restricted access to the study area). No permits were required as *Agriocnemis femina* is not a protected species and the field site is not protected. Moreover, this research did not involve the utilisation of genetic resources that fall within the scope of the global Nagoya protocol.

### 2.3 Parasite prevalence and intensity

We captured damselflies with insect catching nets (dimensions: 1260 mm handle, 456 mm diameter hoop, 81 cm long net bag) while walking along the edge of the water body and adjacent grasslands. We conducted fieldworks between 08:00 and 10:00 h when individuals were most active, and condition were favourable for field work (Paul et al., 2022). For each sampling days we covered the study area and spent approximately 1 h for collecting damselflies. During entire study period, we surveyed for 23 days covering all seasons. After capturing a damselfly, we recorded its sex (male and female), and the developmental stage of females (immature females are red and mature females are green) while male developmental status cannot be determined precisely under field conditions (Khan, 2020; Paul et al., 2022) (See Supplementary file). We examined the damselfly's dorsal and ventral thorax, and abdomen for parasites and if present, counted the number of parasites. To prevent recapture, we marked their wings with a permanent marker and released them back into the population.

### 2.4 Bioclimatic factors

We collected monthly data for temperature and precipitation for 2021–2023 from the Bangladesh Meteorological Department (BMD) and calculated monthly average temperature (°C) and precipitation (mm) for the surveyed population (Bangladesh Meteorological Department, 2023). We conducted statistical analysis utilising monthly climatic data for two reasons. Firstly, the daily climate data were not available for analysis. Secondly, we believe that monthly climatic data better represent seasonal patterns in the study region, whereas daily data could be fluctuating.

### 2.5 Statistical analyses

We applied the DurgaDiff function of the Durga R package to determine mean differences and parasite prevalence and parasite intensity between seasons (Khan & McLean, 2023). 95% confidence intervals of mean difference were calculated by bootstrapping 1000 times. We applied a generalised linear mixed models (GLMMs) to identify the effect of temperature and precipitation on parasite prevalence and intensity. We fitted the GLMM model with parasite prevalence as the response variable, temperature and precipitation as fixed effects and sampling year as a random factor. We further applied generalised linear model (GLM) with a quasipoisson distribution with parasite intensity as the response variable and temperature and precipitation as fixed effects. We analysed all data in R version 4.0.3 (R Core Team, 2020) using packages ‘lme 4’ (Bates et al., 2014), ‘MuMIn’ (Barton, 2010), ‘performance (Lüdecke et al., 2021)’ and ‘Durga’ (Khan & McLean, 2023).

## 3 RESULTS

A total of 2846 individuals of *Agriocnemis femina* were sampled of which 10.6% were parasitised (Table S1, Supplementary file). Parasite prevalence was highest in winter (17.3%) and lowest in summer (4.5%). On average a parasitised individual carried three parasites (range: 1–19). Parasite intensity was greatest in autumn (3.10 parasites/damselfly) and lowest in spring (1.92 parasites/damselfly).

**FIGURE 1:**
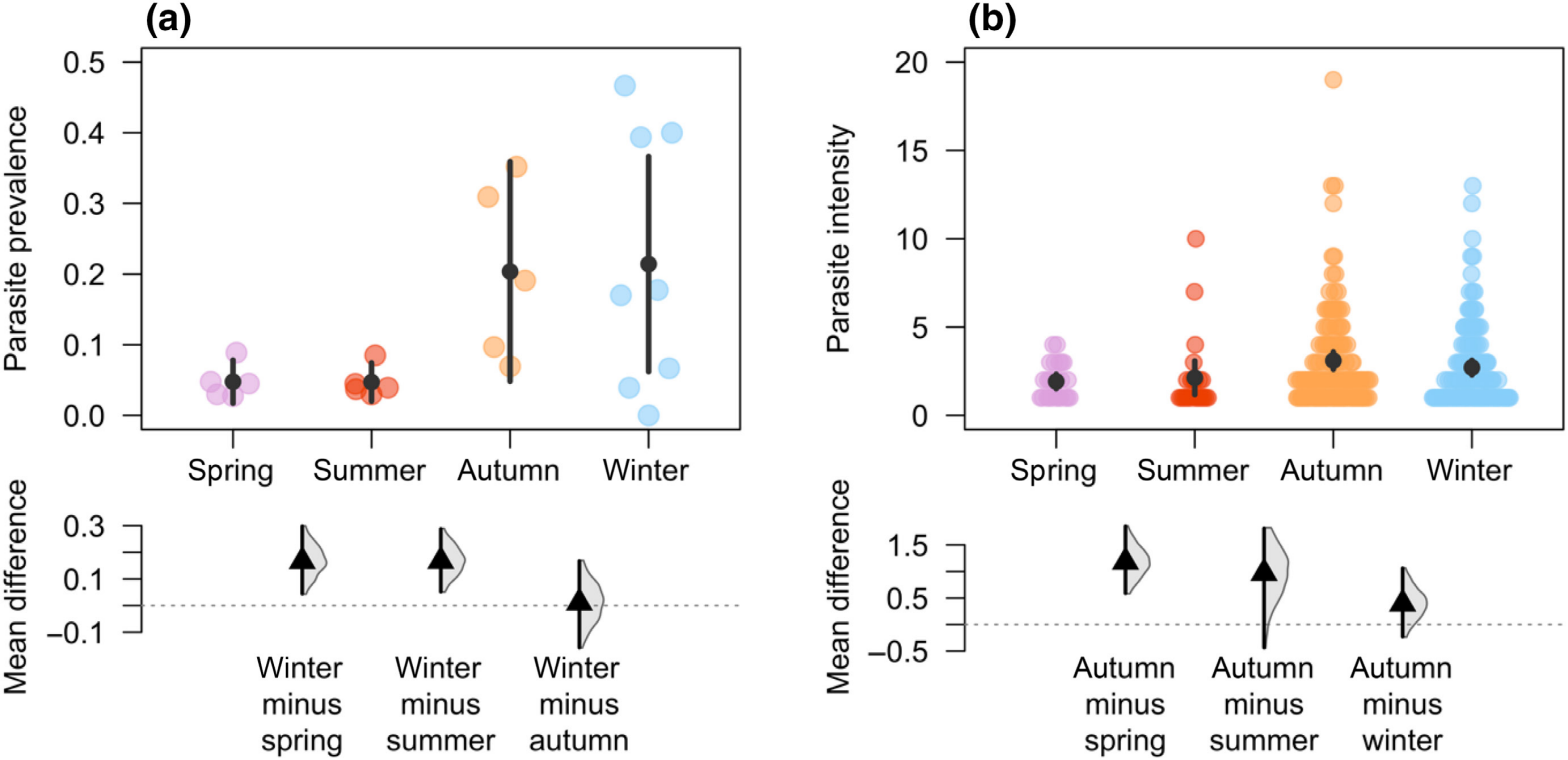
Seasonal variation of parasite prevalence and intensity in *Agriocnemis femina* damselflies. (a) Parasite prevalence and (b) parasite intensity across four seasons. In the upper panel, black circle represents mean and vertical bar represent confidence interval of parasite prevalence (a) and parasite intensity (b) in difference seasons. In the upper pane, each coloured circle represents a sampling event (a) and in (b) represents a parasitised damselfly. The effect size (mean differences) in parasite prevalence and in parasite intensity between seasons is shown in the lower panel where triangle represent mean difference, vertical line represents 95% confidence interval of mean difference and half violin represent density of bootstrapped mean difference.

Parasite prevalence was greater in winter compared to spring (mean difference = 0.166, 95% CI [0.053, 0.304]; Figure 1a) and summer (mean difference = 0.167, 95% CI [0.050, 0.295]; Figure 1a). But parasitism did not differ between winter and autumn (mean difference = 0.010, 95% CI [−0.144, 0.193]; Figure 1a). Parasite intensity was higher in autumn compared to spring (mean difference = 1.180, 95% CI [0.634, 1.839]; Figure 1b) and summer (mean difference = 0.970, 95% CI [−0.532, 1.745]; Figure 1b), but there was no difference in parasite intensity between autumn and winter (mean difference = 0.392, 95% CI [−0.187, 1.071]; Figure 1b).

**FIGURE 2:**
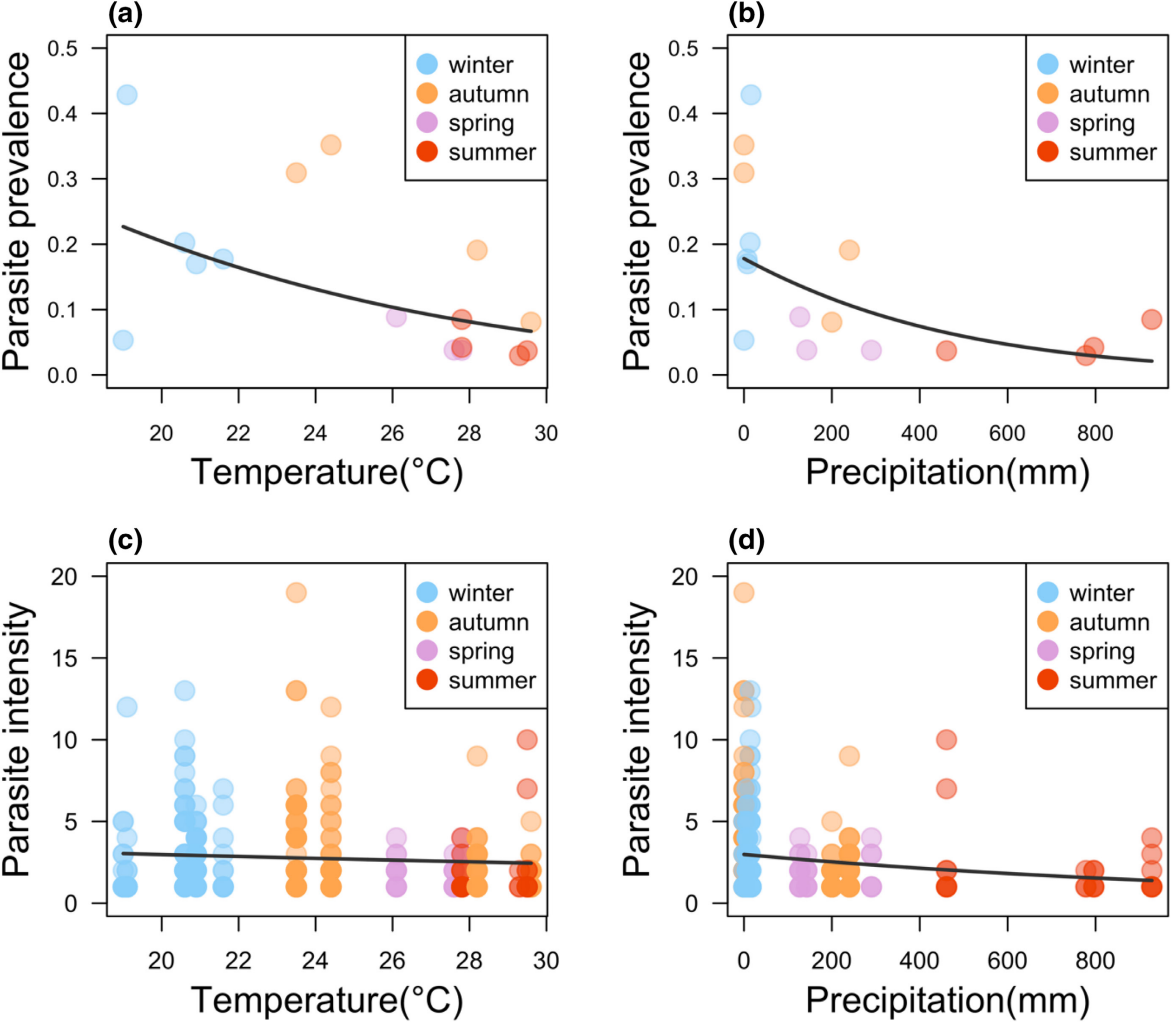
Correlation of parasite prevalence and intensity with temperature and precipitation in *Agriocnemis femina* damselflies. Correlation of parasite prevalence with mean monthly temperature (a) and mean monthly precipitation (b). Correlation of parasite intensity with mean monthly temperature (c) and mean monthly precipitation (d) Each circle in (a) and (b) represents a sampling event and in (c) and (d) represents a parasitised damselfly. The fitted lines in each figure represent overall trend of data points.

Parasite prevalence was negatively related to mean monthly temperature (GLMM: estimate = −0.391 ± 0.089, *z* = −4.375, *p* < .00001; *R* ^2^ = 0.098; Figure 2a) and mean monthly precipitation (GLMM: estimate = −0.475 ± 0.110, *z* = −4.320, *p* < .00001; *R* ^2^ = 0.098; Figure 2b). Parasite intensity was negatively correlated only with precipitation, (GLM: estimate = −0.303 ± 0.126, *t* = −2.398, *p* = .017; Partial *R* ^2^ = 0.018; Figure 2d) but not with temperature (GLM: estimate = 0.051 ± 0.074, *t* = 0.692, *p* = .489; Partial *R* ^2^ = 0.001; Figure 2c).

## 4 DISCUSSION

Climatic variables, such as temperature or precipitation, influence insect physiology and host–pathogen interactions, which might result in differential levels of parasitism across seasons (Fecchio et al., 2019; Gehman et al., 2018; Ovadia & Schmitz, 2004; Powell et al., 2007; Yourth et al., 2002). In our study, we provided strong evidence that water mite prevalence and intensity in damselflies vary across seasons, with higher rates of infection during cooler months (winter and autumn) compared to hotter months (spring and summer). We further showed that, parasite prevalence was greater when temperatures were cooler and rainfall was lower, while parasite intensity was greater with lower rainfall.

The higher prevalence and intensity of parasitism in autumn and winter months compared to spring and summer could stem from an increased susceptibility of damselflies to parasitic infections (Blanford et al., 2003; Robb & Forbes, 2005). In colder months, larval growth rate (De Block & Stoks, 2003) and development time are longer (Norling, 2018; Pritchard, 1989; Trottier, 1971) and larvae and adults are less active (De Block & Stoks, 2003), which might increase the exposure of damselflies to parasites (Forbes & Baker, 1991; Nagel et al., 2009). Thus, water mites might have more time to find a host and engorge (Leung et al., 1999), thereby increasing parasitism in colder months (Leung et al., 1999; Nagel et al., 2009).

Even though, hotter seasons (summer and spring) with higher temperature provide ideal developmental conditions for invertebrates, such as damselflies and water mites (Batzer & Boix, 2016), we observed lower parasitism at higher temperatures. This could be because damselflies mount a greater immune response (encapsulation rate) to infection at higher temperatures (Robb & Forbes, 2005). Accordingly, parasitism in *Coenagrion puella* damselflies was lower in warmer seasons compared to the cooler seasons (Hassall et al., 2010).

Furthermore, the lower rate of parasitism in spring and summer compared to winter could arise because of the impact of the subtropical monsoon in the northeast region of Bangladesh which brings heavy rainfall (on average 695.33 mm during wet and hot summer months) (Fick & Hijmans, 2017). Additionally, our low altitude study area (altitude = 10 m) receives water from the adjacent Meghalaya Hills (altitude = 1961 m), which often experience one of the highest average rainfalls in the world (Barman et al., 2021; Deb et al., 2013; Murata et al., 2007). As a consequence, the study area is frequently flooded (Murata et al., 2007). We argue that the flash flooding probably diluted the density of water mites in the small ponds, similar to a previous study that observed water mite abundance in the tropical river Ganga being greater in the winter months compared to the monsoon months (Rana et al., 2023). Similarly, lower parasitism during high rainfall was also recorded in other aquatic and semi‐aquatic organisms such as in fish (*Cirrhinus mrigala*) and snails (*Elimia proxima*) (Majumder et al., 2015; Zemmer et al., 2017). Conversely, reduced precipitation probably increases parasitism by increasing damselflies' susceptibility and also by increasing the concentration of water mites in water bodies (Shearer & Ezenwa, 2020; Smith et al., 2010).

Our study provides evidence that parasitism in a subtropical study site increases during cooler and drier seasons. Under ongoing anthropogenic climate change, tropical, subtropical and mediterranean regions are expected to experience climatic extremes and seasonal instability, which could stress insects such as damselflies, making them even more vulnerable to parasitism (Day, 2006; Rouault et al., 2006; Salcido et al., 2020). Therefore, we predict that parasitism might increase in aquatic and semi‐aquatic insects especially in tropical, subtropical and mediterranean regions.

Already, odonates with lentic habitats are threatened because climate change induced temperature and rainfall patterns, for example, increase in arid conditions may cause habitat loss (Cerini et al., 2020). Habitat may also be altered due to anthropogenic activities driven by changes in land use, for example, urbanisation and agricultural expansions. Our study highlights that, in addition to habitat loss, climate change‐induced increase in parasitism might further exacerbate odonate fitness and contribute to local extinctions—a research avenue requires additional consideration.

### 4.1 Statement of diversity and inclusion

We believe and support equity, diversity and inclusion in science and everywhere (Rößler et al., 2020). The authors come from different nationalities and cultural backgrounds (Bangladesh, Austria and Australia). They represent different career stages (Masters student, Early career researcher and Professor). One or more of the authors self‐identifies as a member of the LGBTQI+ community and represents ethnic as well as religious minority in science. We actively maintained gender balance while citing scientific articles.

## AUTHOR CONTRIBUTIONS

**Shatabdi Paul:** Conceptualization (supporting); data curation (lead); formal analysis (equal); funding acquisition (lead); methodology (equal); project administration (equal); validation (equal); visualization (equal); writing – original draft (lead). **Mostakim Rayhan:** Data curation (supporting); project administration (supporting). **Marie E. Herberstein:** Investigation (equal); resources (equal); supervision (equal); validation (equal); writing – review and editing (equal). **Md Kawsar Khan:** Conceptualization (lead); data curation (supporting); formal analysis (equal); investigation (equal); methodology (equal); project administration (equal); supervision (lead); validation (equal); writing – original draft (supporting); writing – review and editing (lead).

## CONFLICT OF INTEREST STATEMENT

The authors declare no conflict of interest.

## Supporting information


Data S1.

Table S1.

## Data Availability

All data, code for analysis and data visualisation are deposited in Figshare. https://figshare.com/articles/dataset/_b_Cooler_and_drier_conditions_increase_parasitism_in_subtropical_damselfly_populations_b_/24182859.
