# Supplementary material for: Cooler and drier conditions increase parasitism in a subtropical damselfly population
Source: Ecol Evol. 2024 Jan 31;14(2):e10897. doi: 10.1002/ece3.10897 (PMC10828727; doi:10.1002/ece3.10897)
Supplement: Supplementary file 1 — Data S1. [file ECE3-14-e10897-s001.docx]

**Supporting Information**

**
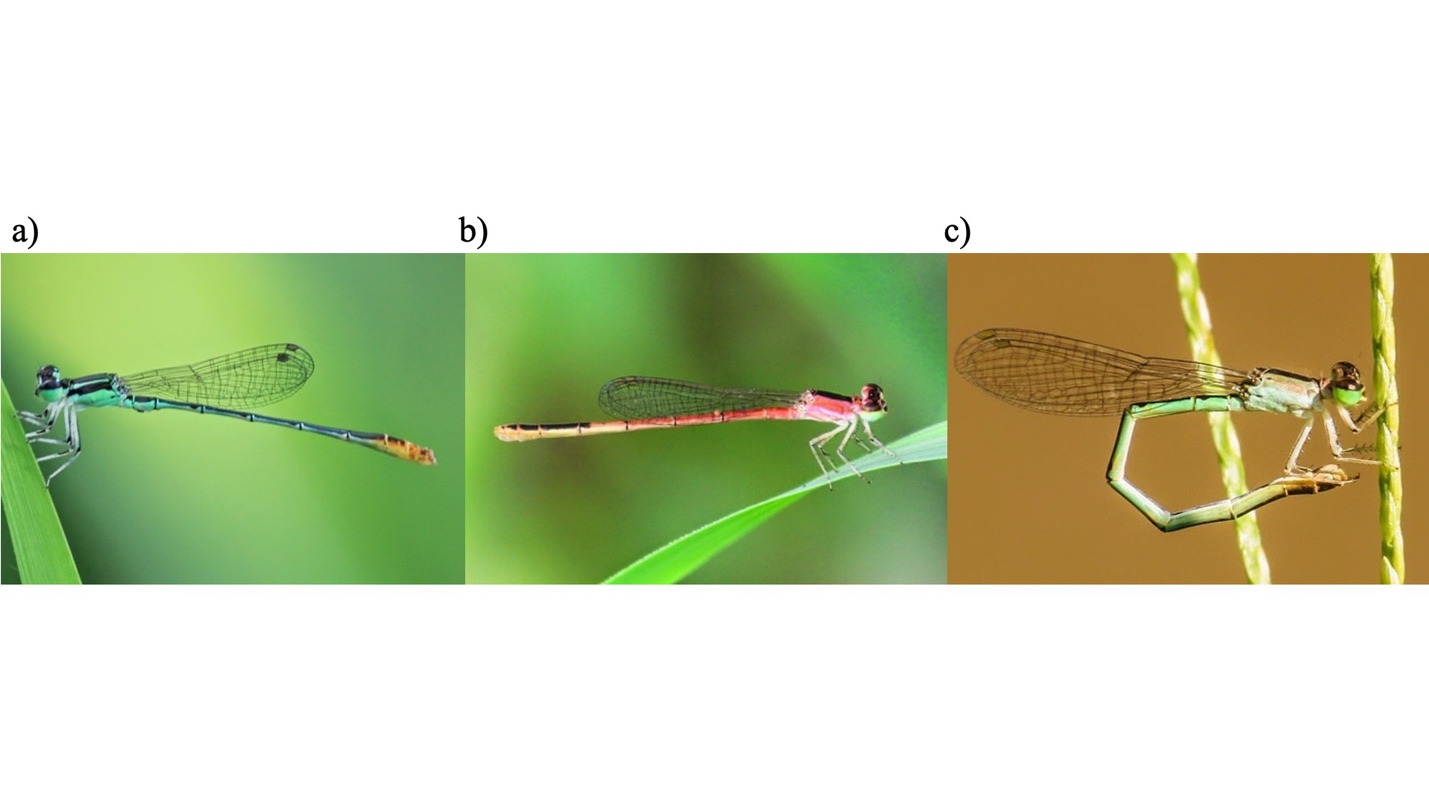
**

**Fig S1. Photographs of male, immature and mature females of *Agriocnemis femina* respectively.** (a) Photograph of a male *Agriocnemis femina* (b) an immature female *Agriocnemis femina* (c) a mature female *Agriocnemis femina.*

1. b)


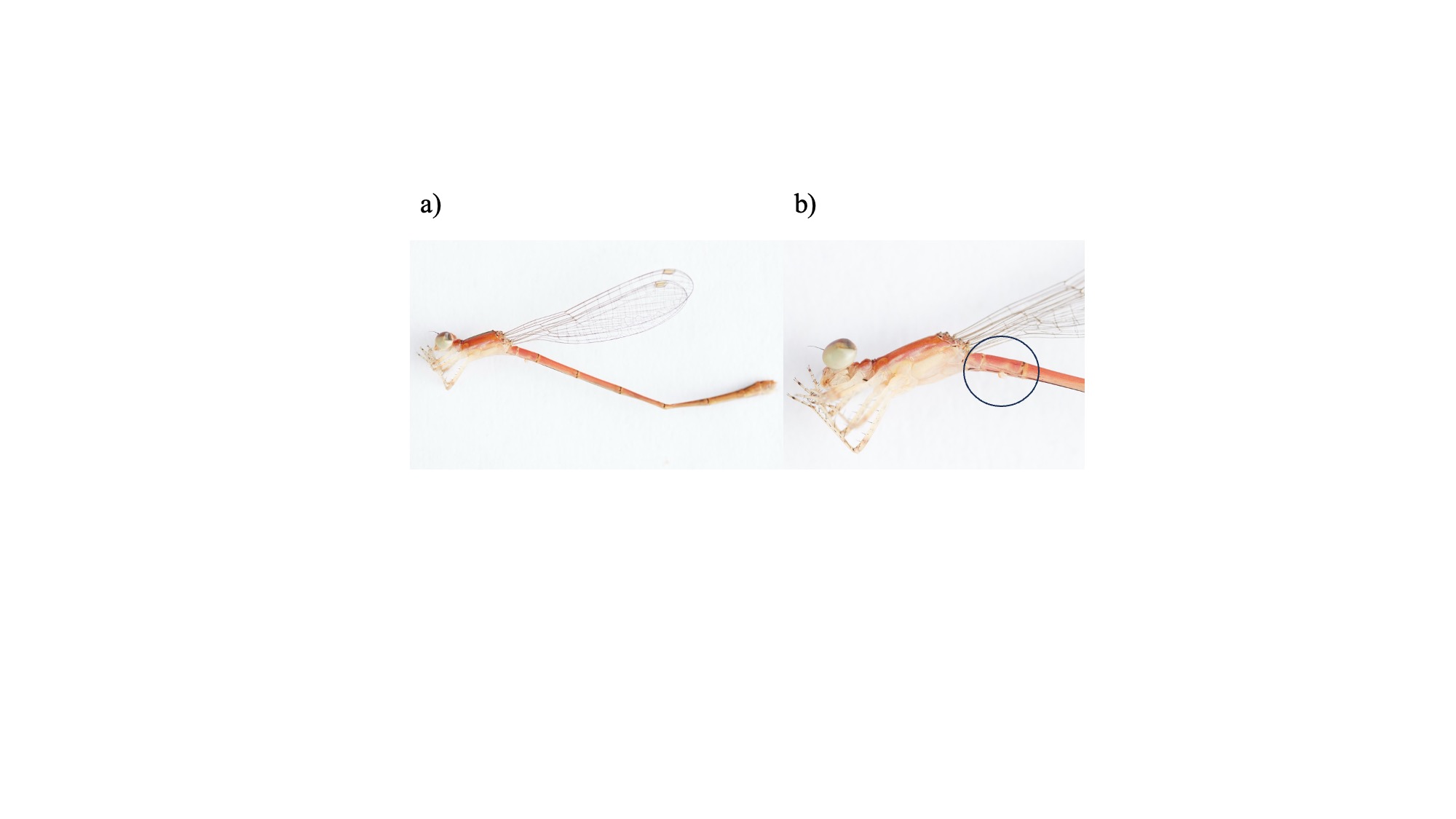


**Fig S2. Photographs of a parasitised *Agriocnemis femina* damselfly and a closeup of water mite parasite respectively.** (a) Photograph of a parasitised immature female of *Agriocnemis femina* (b) a close up of water mite attached on the ventral side of the abdomen of immature female damselfly.

**Generalized linear models**

We used generalized linear mixed models (GLMMs) with binomial distribution to determine variation in prevalence between sampling seasons. We fitted GLMMs with parasite infection status (parasite = 1, no_parasite =0) as a response variable, the interactions between season and sex as fixed effects and sampling year as a random factor. There was no effect of the interaction between sex and season therefore sex was omitted from the final model. We then applied a generalized linear model (GLM) with a quasipoisson distribution to test whether parasite intensity differs across seasons.

**Results**

Parasite prevalence was greater in winter compared to spring (GLMM: estimate = -1.402 ± 0.226, z = -6.186, P < 0.00001; R^2^ = 0.087; Fig 1a). Similarly, compared to summer parasite prevalence was greater in winter (GLMM: estimate: -1.731 ± 0.206, z = -8.384, P < 0.00001; R^2^ = 0.087; Fig 1a). Our data did not provide any evidence that parasitism varied between winter and autumn (GLMM: estimate = 0.018 ± 0.155, z = 0.118, P = 0.906; R^2^ = 0.087; Fig 1a).

Intensity was higher in autumn compared to spring (GLM: estimate = -0.479 ± 0.225, t = -2.121, P = 0.034; R^2^ = 0.034; Fig 1b). Again, the data showed that parasite intensity was greater in autumn compared to summer (GLM: estimate = -0.375 ± 0.223, t = -1.676, P = 0.094; R^2^ = 0.034; Fig 1b). However, we did not find any difference in parasite intensity between autumn and winter (GLM: estimate = -0.135 ± 0.10878, t = -1.243, P = 0.214; R^2^ = 0.034; Fig 1b).

Parasite prevalence was not associated with mean monthly temperature within winter and summer season respectively (GLMM: estimate = 0.53± 0.392, z = 1.351, P = 0.177; R^2^ = 0.005; Fig S3(a), GLMM: estimate = -0.953± 0.842, z = -1.132, P = 0.257; R^2^ = 0.013; Fig S3(d)). However, within autumn and summer seasons, monthly temperature was negatively correlated with parasite prevalence (GLMM: estimate = -0.949± 0.139, z = -6.801, P < 0.00001; R^2^ = 0.122; Fig S3(b)). Parasite prevalence was variation was negatively correlated with monthly temperature of spring season, (GLMM: estimate = -2.081± 0.848, z = -2.454, P = 0.014; R^2^ = 0.047; Fig S3(c)).


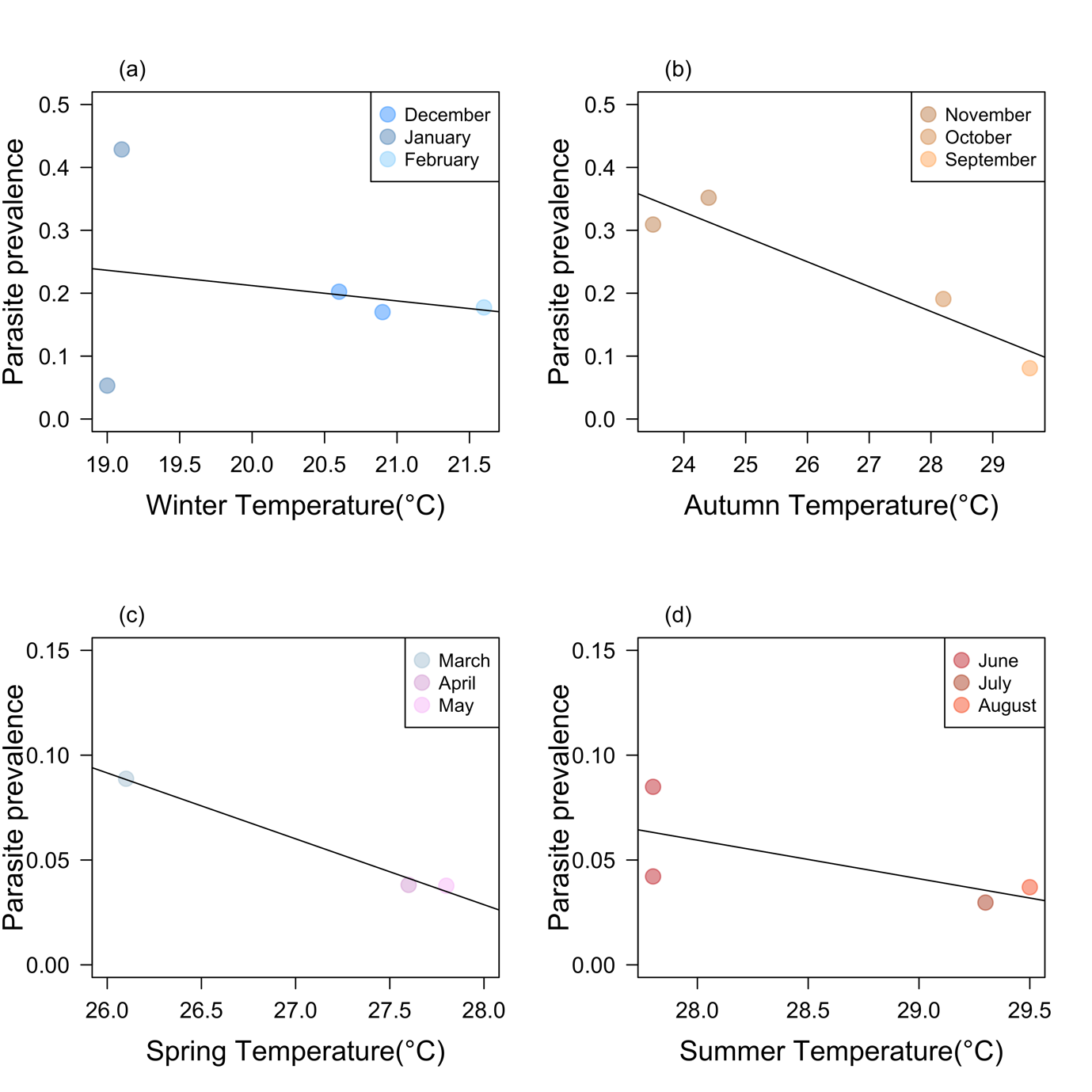


**Fig S3. Correlation of parasite prevalence in *Agriocnemis femina* damselflies with monthly temperature within each of the four seasons.** Correlation of parasite prevalence with mean monthly temperature of winter season (a), of autumn season (b), of spring season (c), of summer season (d). Each circle in (a), (b), (c), and (d) represents a sampling event. The fitted lines in each figure represent overall trend of data points.
