# Supplementary material for: Cooler and drier conditions increase parasitism in a subtropical damselfly population
Source: Ecol Evol. 2024 Jan 31;14(2):e10897. doi: 10.1002/ece3.10897 (PMC10828727; doi:10.1002/ece3.10897)
Supplement: Supplementary file 2 — Table S1. [file ECE3-14-e10897-s002.docx]

**Table S1: Field sites, locations, and number of total and parasitised *Agriocnemis femina* collected at different seasons for calculating parasite prevalence and intensity.**

| Field site | Location | Season | Month | Total | Parasitised | Intensity range- | Temperature  range (°C) | Precipitation  Range (mm) |
| --- | --- | --- | --- | --- | --- | --- | --- | --- |
|  |  |  |  |  |  |  |  |  |
| SUST gate | 24.911667 N, 91.831944 E | Spring | March-May | 617 | 32 | 1-4 | 26.1-27.8 | 127-290 |
| SUST gate | 24.911667 N, 91.831944 E | Summer | June-August | 755 | 34 | 1-10 | 27.8-29.5 | 461-928 |
| SUST gate | 24.911667 N, 91.831944 E | Autumn | September-November | 688 | 134 | 1-19 | 23.5-29.6 | 0-240 |
| SUST gate | 24.911667 N, 91.831944 E | Winter | December-February | 786 | 136 | 1-13 | 19-21.6 | 0-16 |
